# Denervation-related alterations and biological activity of miRNAs contained in exosomes released by skeletal muscle fibers

**DOI:** 10.1038/s41598-017-13105-9

**Published:** 2017-10-16

**Authors:** Rita De Gasperi, Sayyed Hamidi, Lauren M. Harlow, Hanna Ksiezak-Reding, William A. Bauman, Christopher P. Cardozo

**Affiliations:** 1 National Center for the Medical Consequences of Spinal Cord Injury, James J. Peters VA Medical Center, Bronx, NY USA; 2 Medical Service, James J. Peters VA Medical Center, Bronx, NY USA; 3 Department of Medicine, Icahn School of Medicine at Mount Sinai, New York, NY USA; 4 Department of Rehabilitation Medicine, Icahn School of Medicine at Mount Sinai, New York, NY USA; 5 Department of Pharmacologic Science, Icahn School of Medicine at Mount Sinai, New York, NY USA; 6 Department of Neurology, Icahn School of Medicine at Mount Sinai, New York, NY USA; 7 Department of Psychiatry, Icahn School of Medicine at Mount Sinai, New York, NY USA; 8 Friedman Brain Institute, Icahn School of Medicine at Mount Sinai, New York, NY USA

## Abstract

Exosomes are vesicles released by many eukaryotic cells; their cargo includes proteins, mRNA and microRNA (miR) that can be transferred to recipient cells and regulate cellular processes in an autocrine or paracrine manner. While cells of the myoblast lineage secrete exosomes, it is not known whether skeletal muscle fibers (myofibers) release exosomes. In this study, we found that cultured myofibers release nanovesicles that have bilamellar membranes and an average size of 60–130 nm, contain typical exosomal proteins and miRNAs and are taken up by C2C12 cells. miR-133a was found to be the most abundant myomiR in these vesicles while miR-720 was most enriched in exosomes compared to parent myofibers. **Treatment of NIH 3T3 cells with myofiber-derived exosomes downregulated the miR-133a targets proteins Smarcd1 and Runx2, confirming that these exosomes have biologically relevant effects on recipient cells**. Denervation resulted in a marked increase in miR-206 and reduced expression of miRs 1, 133a, and 133b in myofiber-derived exosomes. These findings demonstrate that skeletal muscle fibers release exosomes which can exert biologically significant effects on recipient cells, and that pathological muscle conditions such as denervation induce alterations in exosomal miR profile which could influence responses to disease states through autocrine or paracrine mechanisms.

## Introduction

Skeletal muscle is now recognized as a major secretory organ that releases soluble mediators capable of acting locally or on distant tissues^1^ such as pancreas^2^, adipose tissue^3^ and bone^4^. Recently, it has been recognized that cells can also communicate through the release of exosomes, which are membranous vesicles of about 50–150 nm in diameter that are derived from multivesicular bodies (MVB) during endosome maturation^5^. Exosomes are released into the extracellular space by fusion of the MVB with the plasma membrane^5^. In addition to lipids, exosomes contain proteins, some of which are characteristic of exosomes (tetraspanins, HSP70, Alix and TSG101), while others reflect the proteins synthesized in the cell of origin^5^. Exosomes also contain mRNA and microRNA (miR) cargo which can be transferred to recipient cells and either be translated (mRNAs) or interfere with translation (miRs)^6^. The diverse cargos delivered by exosomes to recipient cells thus have the potential to affect many different biological processes^5^.

Exosomes derived from C2C12 myoblasts or myotubes have been isolated and their protein and miR composition has been analyzed^7–11^. Myotube-derived exosomes influenced myoblast proliferation and differentiation, and exosomal proteins were incorporated into recipient myoblasts^7^. Profiling of miRs within exosomes released by C2C12 myoblasts and myotubes showed that miRs were selectively incorporated^8^. Moreover, miRs within exosomes from myotubes silenced Sirtuin1 in myoblasts^8^. C2C12 derived exosomes were also found to enhance survival and neurite growth of the motor neuron cell line NSC-34, suggesting that muscle could influence motor neuron survival and axon growth^12^. In a mouse model of high-fat diet-induced insulin resistance, exosome-like vesicles isolated from skeletal muscle were taken up by cultured MIN6B1 cells and modulated their gene expression^13^. Moreover, C2C12 derived exosomes were taken up by many organs when injected in mice tail veins^8^. Exosomes secreted by differentiating human skeletal myoblasts were found to induce myogenesis of human adipose-derived stem cells and to enhance regeneration when injected in injured muscle^14^. Collectively these data suggest that exosomes originating from cells of the myogenic lineage may function as autocrine, paracrine or endocrine mediators.

Due to the difficulty in harvesting exosomes from tissues, most of the work to date to characterize muscle exosomes has been conducted using the C2C12 cell line. Studies of exosome-like particles isolated from muscle have not addressed the fact that skeletal muscle contains many cell types in addition to myofibers, such as nerves, blood vessels, connective tissue, and fat. This point raises concerns regarding any of the exosomal findings due to the evident uncertainty regarding the cells of origin. While it is likely that skeletal muscle fibers (myofibers), a syncytium formed by the fusion of tens to hundreds of myocytes, do indeed release exosomes, this possibility has not been directly addressed. In addition, the question of how pathological states alter the cargo of exosomes derived from myofibers has not been investigated. This study determined whether dispersed mouse myofibers release exosomes, how paralysis induced by nerve transection altered the miR cargo of exosomes released by such fibers and whether such exosomes influence biology of recipient cells. Our findings establish that myofibers release exosomes and modify their cargo during pathological conditions, and that such exosomes regulate expression of Smarcd1 (BAF60a), a structural component of the BAF chromatin remodeling complex which has critical roles in myogenesis^15,16^, and Runx2, which is a master regulator of osteogenic lineage progression^17^. The findings have important implications for understanding the mechanisms by which myofibers signal to nearby cells within skeletal muscle, as well other more distant tissues.

## Results

### Cultured Muscle fibers release exosome-like nanovesicles

**Figure 1:**
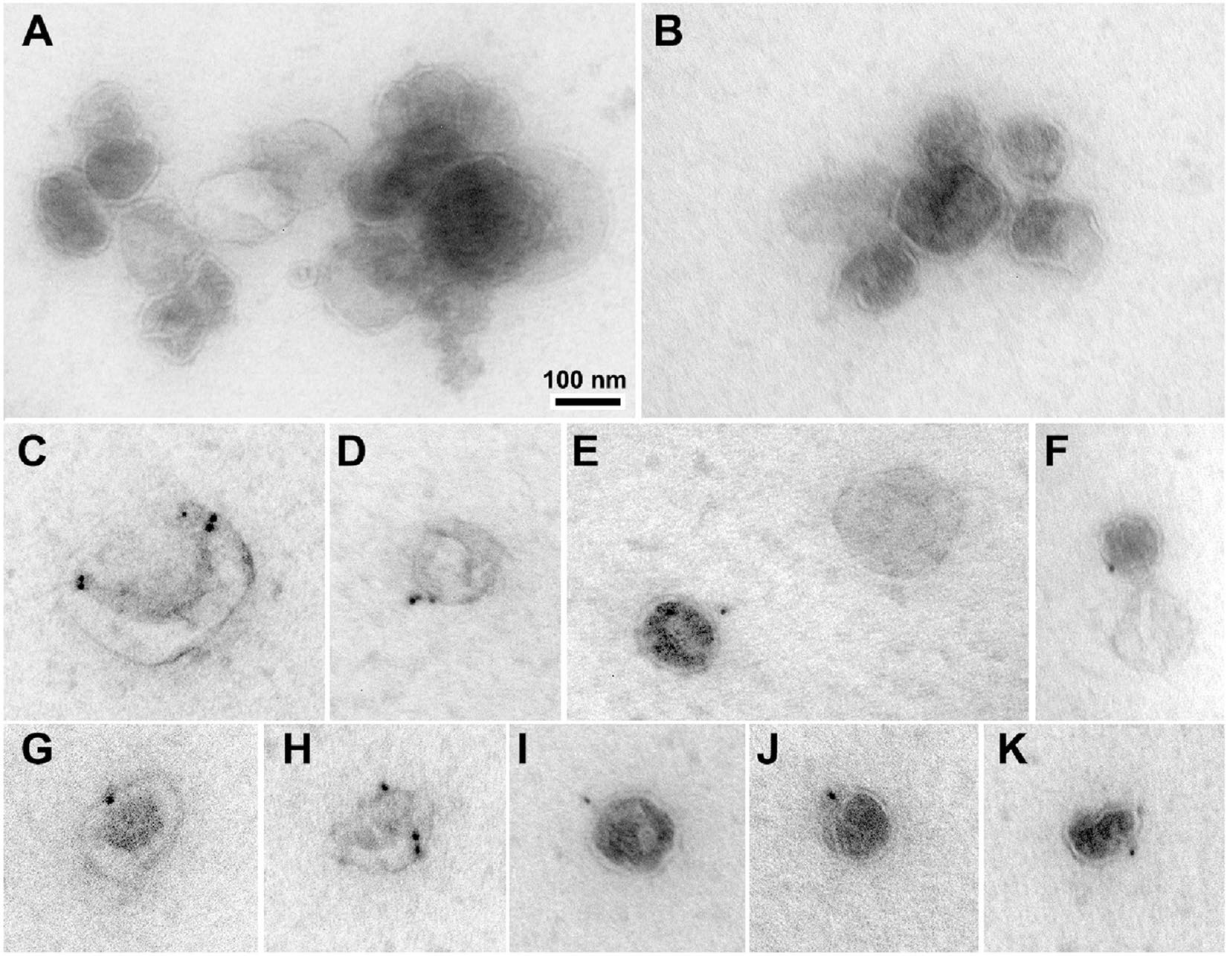
EM characterization of nanovesicles released by dispersed muscle fibers. Dispersed mouse hindlimb muscle fibers were incubated for 48 hours. Nanovesicles were isolated by ultracentrifugation of conditioned medium then stained with uranyl acetate (**A**,**B**) or labeled with 10-nm immunogold particles using antibodies against the exosomal membrane markers CD63 (**C**–**F**), CD81 (**G**–**J**) and CD9 (**K**), and stained with uranyl acetate. Nanovesicles were imaged by electron microscopy. Scale bar: 100 nm.

To determine whether myofibers isolated from the mouse hindlimb muscles, namely soleus, plantaris, gastrocnemius and EDL, release nanovesicles, dispersed myofibers were cultured for 48 hours in exosome-depleted medium. The conditioned media was collected and subjected to sequential centrifugation. The pellet obtained by centrifugation at 100,000 × g was analyzed by electron microscopy (Fig. 1) which revealed particles that varied in size from approximately 60 to 130 nm in diameter and had a bilamellar membrane (A and B). By immunogold staining, the vesicles were labeled by antibodies against CD63 (C–F), CD81 (G–J) and CD9 (K).

**Figure 2:**
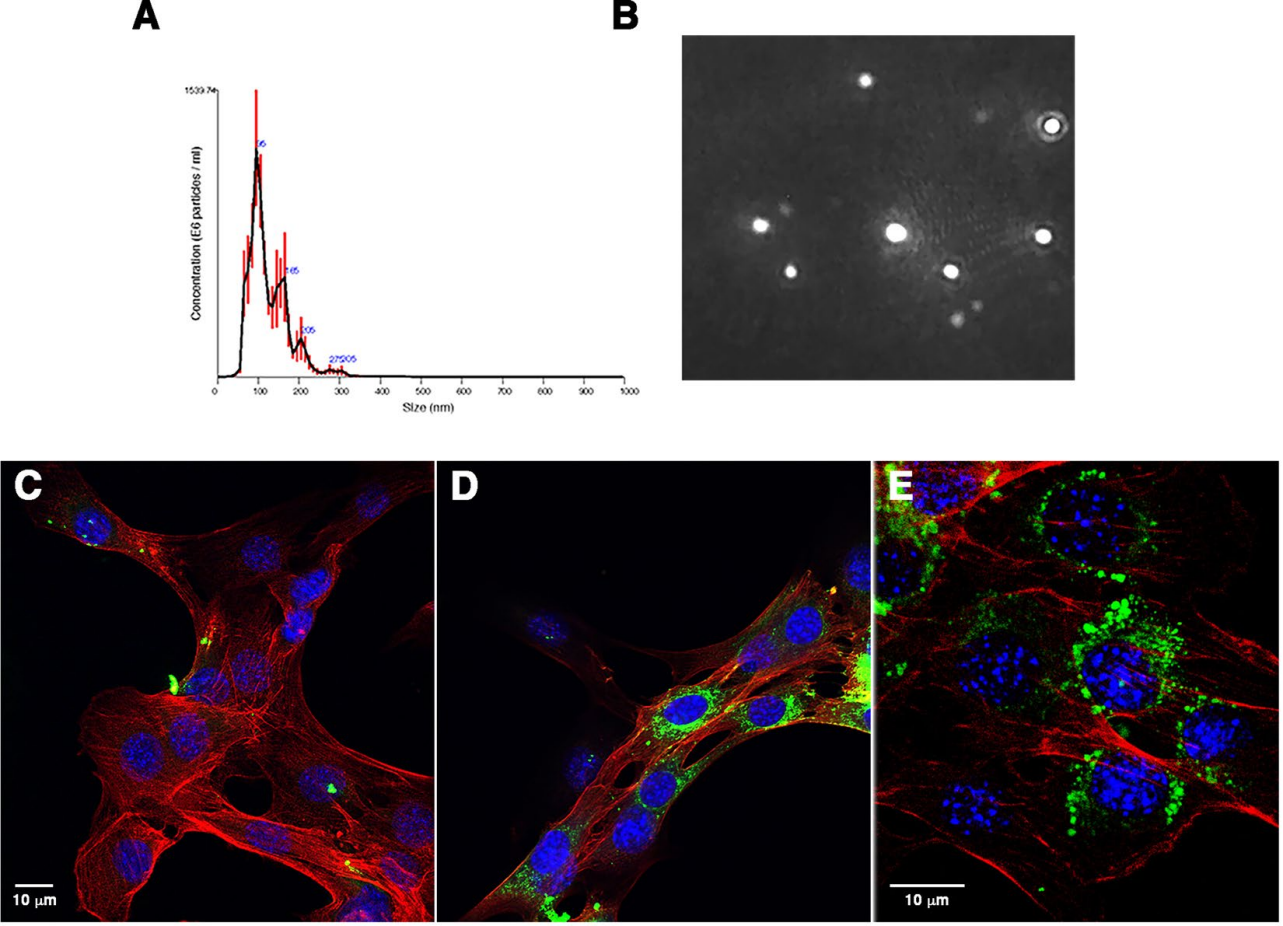
Size distribution and uptake by cells of nanovesicles released by dispersed muscle fibers. Dispersed mouse muscle fibers were incubated for 48 hours, after which nanovesicles were isolated by differential centrifugation. (**A** and **B**) The size distribution and concentration of particles was analyzed by nanoparticle tracking. (**C** and **E**) Nanovesicles were labeled with PKH67 dye and overlaid on cultures of proliferating C2C12 cells. The control cells were treated with an equal volume of dye-treated PBS. After 5 hours, the cells were fixed, stained with phalloidin to visualize cell bodies, counterstained with DAPI to label nuclei and imaged by confocal microscopy. Panel (C) shows control cells incubated in media containing PKH67-treated PBS; panels (D and E) show cells incubated with PKH67-labeled nanoparticles. In these images, nanoparticles are green, phalloidin is red and nuclei are blue (DAPI).

To further analyze the size distribution of these nanovesicles, the particles were subjected to nanoparticle tracking analysis. Approximately 1.9 × 10^9^ vesicles were obtained from the media conditioned by incubation of about 300 myofibers for 48 hours (Fig. 2A and B). Nanovesicles had a mean size of 121 + /− 3.7 nm.

Collectively, the data indicate that dispersed mouse hindlimb muscle fibers release nanoparticles with the size, biochemical markers and biological properties characteristic of exosomes.

### Uptake of labeled nanovesicles by C2C12 myoblasts

A fundamental property of exosomes is that they are taken up by recipient cells thereby delivering their cargo and influencing biological processes^5,6^. To determine whether the nanovesicles isolated from media conditioned by culture with dispersed myofibers were taken up by recipient cells, we treated C2C12 cells with vesicles released by myofibers that had been labeled with the fluorescent dye PKH67. C2C12 myoblasts showed uptake of PKH67-labeled vesicles (Fig. 2D and E) while cells that received the dye-only control had negligible fluorescence (Fig. 2C).

### Influence of denervation on miR profiles in myofibers

Denervation atrophy has been found to result in significant changes in miR expression profiles in muscle tissue including a significant increase in miR-206 levels^18^. To determine miR expression profiles in myofibers and understand how such profiles were altered by denervation, and how miR levels in exosomes released by myofibers differ from those of the parent fiber, miR expression profiles of denervated or sham-denervated EDL myofibers were determined at 7 days post-denervation or sham-denervation using the nCounter mouse miR assay. This technique profiles expression levels of 578 mouse microRNAs. Using a mean of 100 counts in the sham-denervated group as a lower limit for inclusion, data for 58 miRs were evaluated in subsequent analysis (Tables 1 and S1). Expression levels ranged from a low of 100 (miR-148a) to a high of ~21,000 counts (miR-1). The most highly expressed miRs present in myofibers were miR-1, miR-133a, miR-22, miR-378 and miR-720 (Table 1 and S1). When comparing the profiles of miRs from denervated and sham-denervated groups, significant differences were observed for 20 miRs (Table 1 and S1), including decreased expression of miR-1 and 133a and a 25-fold increase in the expression of miR-206 (Table 1).

**Table 1:** nCounter analysis: Significantly altered miRs in denervated fibers

miR	ID	Mean Sham	SD Sham	Mean DN	SD DN	fold-change	P value
mmu-let-7e	MIMAT0000524	400	17	287	68	0.72	0.049
mmu-miR-1	MIMAT0000123	21018	5442	10364	2535	0.49	0.037
mmu-miR-100	MIMAT0000655	399	45	209	67	0.52	0.015
mmu-miR-106a + mmu-miR-17	MIMAT0000385	132	6	171	14	1.29	0.011
mmu-miR-132	MIMAT0000144	124	26	64	10	0.52	0.021
mmu-miR-133a	MIMAT0000145	10120	1261	4685	813	0.46	0.003
mmu-miR-133b	MIMAT0000769	393	48	632	89	1.61	0.015
mmu-miR-143	MIMAT0000247	350	143	108	43	0.31	0.048
mmu-miR-145	MIMAT0000157	620	157	239	28	0.38	0.014
mmu-miR-148a	MIMAT0000516	100	11	67	12	0.67	0.027
mmu-miR-16	MIMAT0000527	927	96	648	130	0.70	0.040
mmu-miR-188-5p	MIMAT0000217	131	5	111	12	0.84	0.049
mmu-miR-206	MIMAT0000239	649	112	16354	5002	25.19	0.006
mmu-miR-27a	MIMAT0000537	460	31	849	105	1.84	0.004
mmu-miR-29b	MIMAT0000127	212	12	316	59	1.49	0.040
mmu-miR-29c	MIMAT0000536	1467	253	740	137	0.50	0.012
mmu-miR-30a	MIMAT0000128	759	191	435	53	0.57	0.047
mmu-miR-30b	MIMAT0000130	270	12	170	38	0.63	0.013
mmu-miR-30d	MIMAT0000515	319	40	181	32	0.57	0.010
mmu-miR-365	MIMAT0000711	272	81	127	31	0.46	0.044
mmu-miR-378	MIMAT0003151	3154	339	1982	271	0.63	0.009

Data are mean, normalized counts for 3 Sham and 3 Denervated animals. Fold-change was calculated as mean counts for the denervated group/mean counts for the sham group. SD, standard deviation.

### Relative abundance of selected miRs in exosomes from myofibers

**Figure 3:**
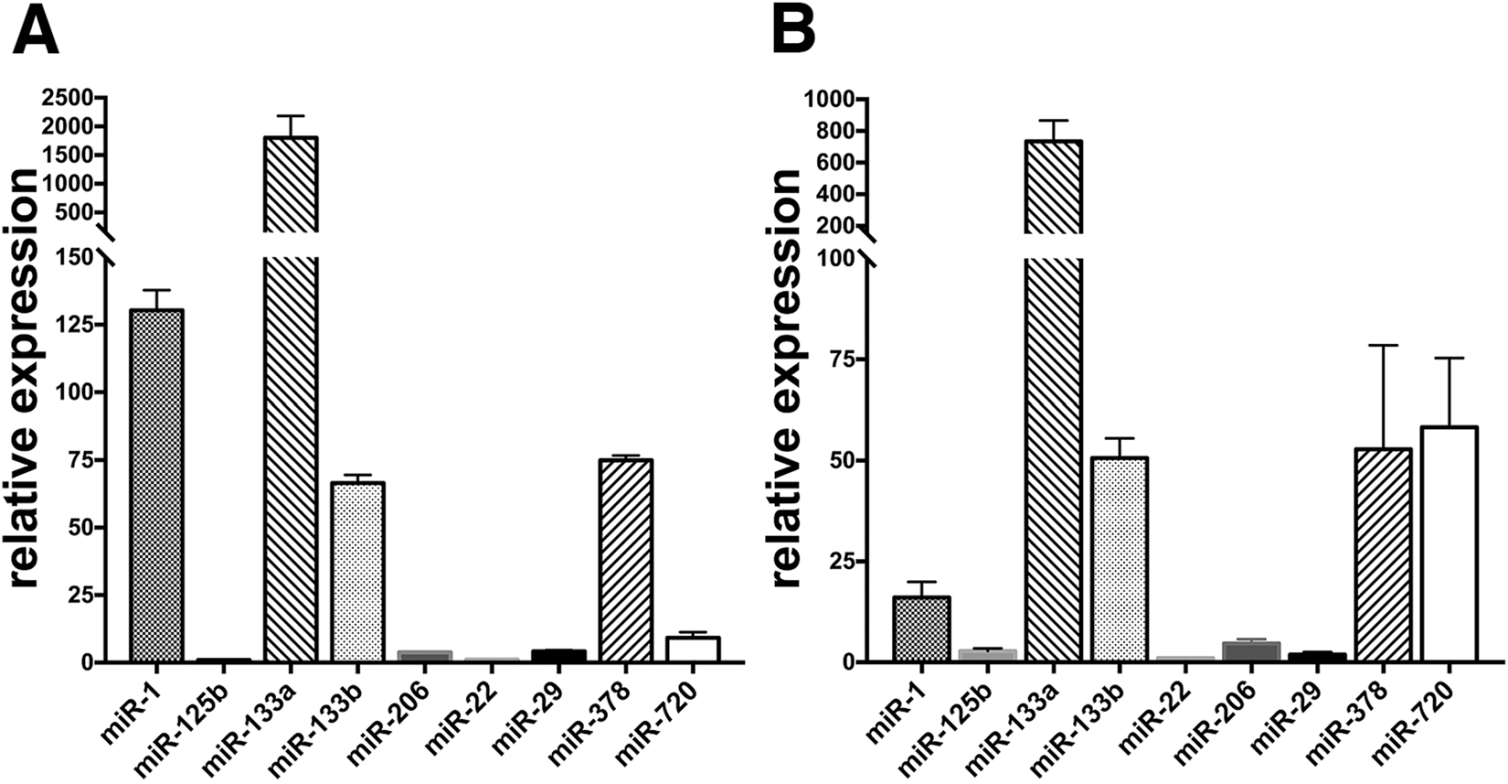
Expression of myomiRs and other selected miR targets in fiber-derived exosomes. Fibers derived from mixed calf muscle were cultured for 48 hours in exosome- free medium. RNA was isolated from fibers (**A**) and corresponding nanovesicles (**B**) and miR expression analyzed by qPCR. The expression is relative to that of miR-22 which was the lowest expressed among the targets tested. The data are from three independent fiber/exosomes preparations.

Based on the above results, qPCR was used to investigate the expression of myomiRs and other selected miR targets in nanovesicles and parent myofibers from control muscle (Fig. 3A and B). This analysis showed that miRs could be detected in fiber-derived nanovesicles and that miR-133a and miR-1 were highly expressed in exosomes relative to miR-22 (Fig. 3B), a miR which was present at similar levels in control and denervated myofibers based on counts observed by nCounter analysis (Table S1). Compared to parent myofibers, miR-720 was enriched in exosomes while the opposite was true for miR-1 (Fig. 3A and B).

### Alteration in miR expression in exosomes from denervated fibers

**Figure 4:**
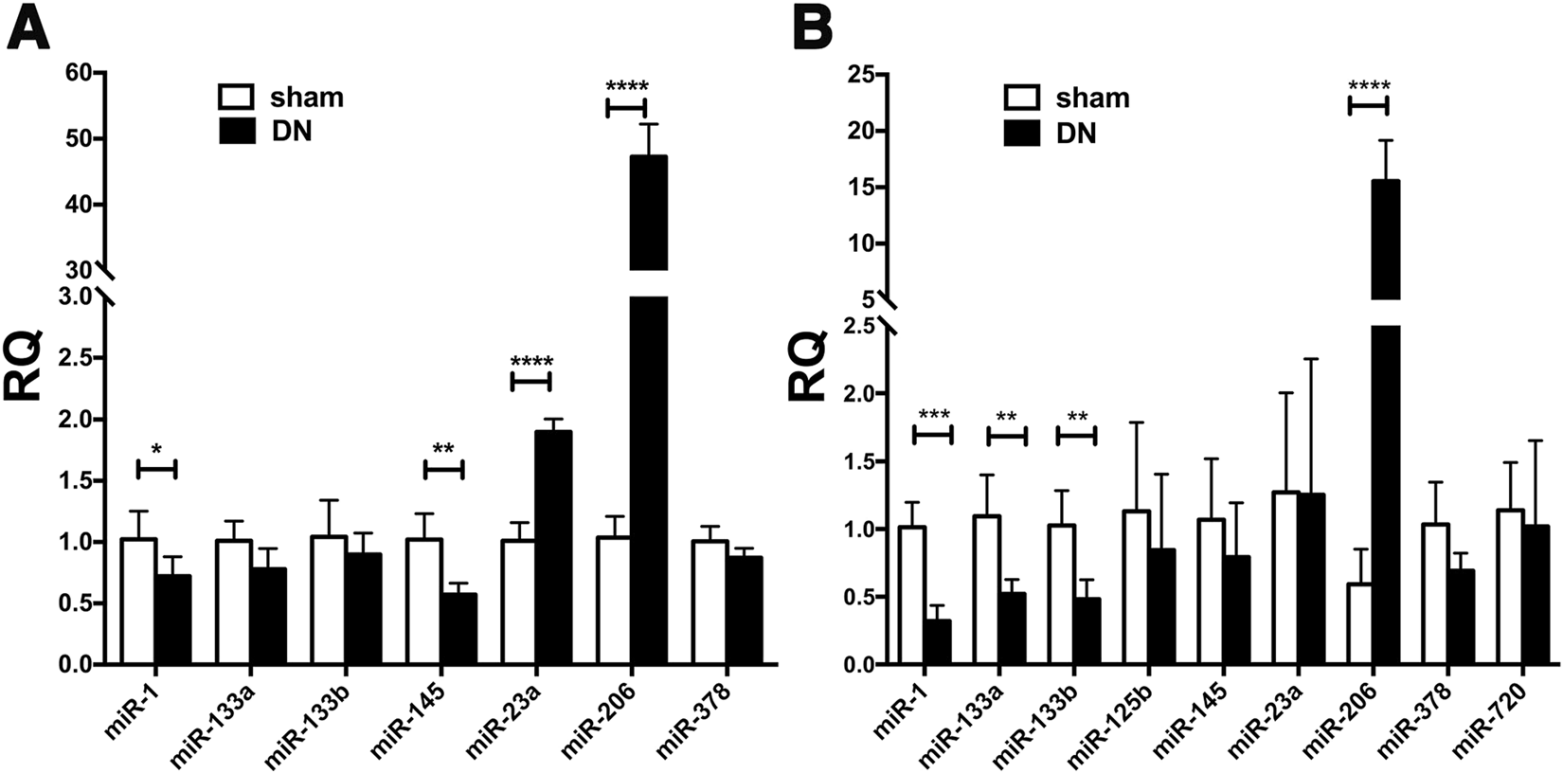
qPCR evaluation of the effect of denervation on miRs expression. Dispersed muscle fibers were isolated from mouse EDL muscle 7 days after sciatic nerve transection or sham transection and maintained in culture in exosome-free medium for 48 hours. RNA was extracted from dispersed fibers (**A**) and corresponding nanovesicles preparations (**B**) and miRs expression levels were determined by qPCR. Data are mean values ± SD for 5 animals per group except for the exosomes produced by denervated fibers (n = 4). *p < 0.05; **p < 0.01; ***p < 0.001.

Exosomes released by the denervated EDL myofibers also showed a marked increase in miR-206 levels (about 15-fold) and a reduction in the levels of miR-1 (Fig. 4B), as in myofibers (Fig. 4A). In contrast to myofibers, exosomes showed significantly reduced levels of miR-133a and miR-133b, a change which was not detected in the corresponding fibers (Fig. 4A and B).

### mRNA targets of miRs altered in exosomes from denervated fibers

To understand potential biological effects of the changes in miR profiles observed, experimentally confirmed mRNA targets of miRs that were altered in exosomes produced by denervated myofibers were identified by Ingenuity Pathways Analysis. This analysis revealed 214 mRNA targets which included growth factors and their receptors (IGF-1, IGF-1R, CTGF, EGFR), molecules involved in calcium-calmodulin signaling (Calm1, NFATC4) and transcriptional regulation (MEF2A, HDAC4, RUNX2) (Tables 2 and S2). Ingenuity Pathways Analysis was then used to identify canonical pathways represented by these mRNAs. The most significantly represented pathways were Myc, IGF-1 and calcium signaling (Tables 2 and S2). Also significant were STAT3, a downstream mediator of IL-6 signaling, and PTEN, which exerts negative feedback on IGF-1, insulin and other pathways downstream of receptor tyrosine kinases. An analysis of upstream regulators of these pathways showed that the most significant correlations were with myomirs 1 and 133, but that TGF-β and estradiol signaling were also highly significantly related.

**Table 2:** Targets of dysregulated miRs present in exosomes released by denervated fibers.

Ingenuity Canonical Pathways	-log(p-value)	Molecules
Myc Mediated Apoptosis Signaling	3.19E + 00	YWHAQ, CASP9, IGF1, IGF1R, BCL2
IGF-1 Signaling	3.18E + 00	YWHAQ, CTGF, CASP9, IGF1, IGF1R, SRF
Calcium Signaling	2.70E + 00	HDAC4, MEF2A, TPM3, TPM1, NFATC4, TPM4, TPM2
Purine Ribonucleosides Degradation to Ribose-1-phosphate	2.58E + 00	PNP, PGM2
Huntington’s Disease Signaling	2.55E + 00	HDAC4, CASP9, IGF1, BDNF, IGF1R, POLR2K, DNAJB1, EGFR
UDP-N-acetyl-D-galactosamine Biosynthesis II	2.30E + 00	GNPNAT1, GNPDA2
STAT3 Pathway	2.23E + 00	PIM1, IGF1R, EGFR, BCL2
PTEN Signaling	2.18E + 00	CASP9, INPP5F, IGF1R, EGFR, BCL2
Estrogen-Dependent Breast Cancer Signaling	2.14E + 00	IGF1, IGF1R, ESR1, EGFR
Cardiac Hypertrophy Signaling	2.04E + 00	HAND2, IGF1, RHOA, IGF1R, SRF, MEF2A, NFATC4
PEDF Signaling	2.01E + 00	BDNF, RHOA, SRF, BCL2
Hepatic Fibrosis/Hepatic Stellate Cell Activation	2.01E + 00	MET, CTGF, IGF1, IGF1R, EGFR, BCL2
Xanthine and Xanthosine Salvage	2.00E + 00	PNP
Chondroitin Sulfate Biosynthesis (Late Stages)	1.90E + 00	UST, CHSY1, CHST11
Clathrin-mediated Endocytosis Signaling	1.86E + 00	MET, IGF1, PICALM, GAK, ITGB4, F2
Epithelial Adherens Junction Signaling	1.82E + 00	MET, NOTCH2, NOTCH3, RHOA, EGFR
Semaphorin Signaling in Neurons	1.81E + 00	MET, RHOA, NRP1
Cholecystokinin/Gastrin-mediated Signaling	1.74E + 00	RHOA, SRF, MEF2A, EGFR
Guanine and Guanosine Salvage I	1.71E + 00	PNP
Adenine and Adenosine Salvage I	1.71E + 00	PNP

The top 20 most significant pathways represented by validated targets for dysregulated miRs present in exosomes released by denervated fibers are listed. A complete listing of significant pathways is shown in Table S2.

### Myofiber-derived exosomes downregulate Smarcd1 and Runx2

**Figure 5:**
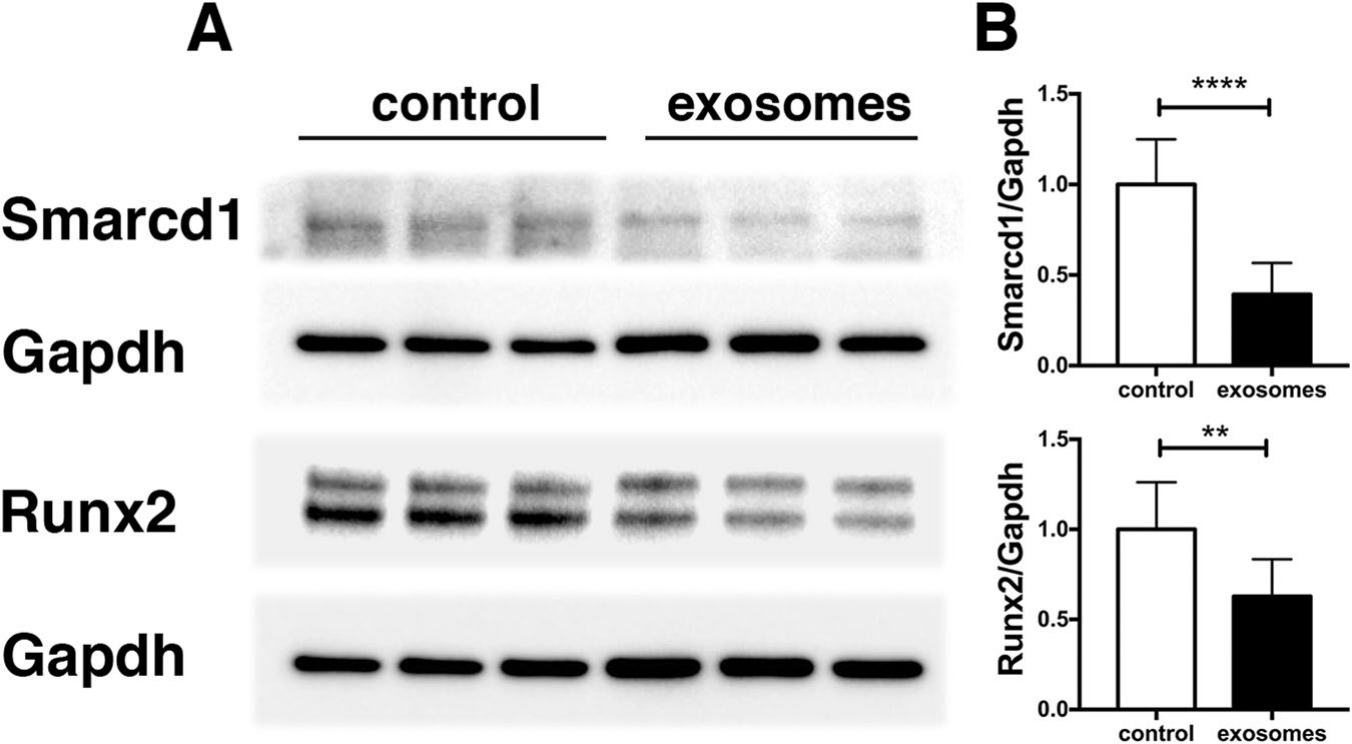
Fiber-derived exosomes modulate Smarcd1 and Runx2 expression. NIH3T3 grown in exosome- depleted medium were treated with fiber-derived exosomes (4 μg/ml) or PBS for 48 hours. The expression of Smarcd1 and Runx2 was analyzed by Western blot. Representative blots are shown in panel (A). Panel (B) shows the quantitation of Smarcd1 and Runx2 expression relative to Gapdh for three separate experiments, each with 3 wells per condition for a total of 9 samples for each condition. **p < 0.005, ****p < 0.0001. Full-length blots are presented in Supplementary Figure S1.

To determine whether exosomes released by myofibers exert biological effects on recipient cells, we tested whether treatment of NIH 3T3 cells with exosomes released by myofibers from healthy muscle altered protein levels of either of two known targets of miR-133a, Smarcd1, and Runx2. Smarcd1^16^ participates in programs that specify cardiac and skeletal muscle development^15,16^. Runx2 is a master regulator of osteogenic lineage progression^17^. These targets were chosen for analysis because, as indicated above, miR-133a is the most abundant miR found in fiber-derived exosomes in our analysis and NIH 3T3 cells are ideal recipient cells to study the biological effects of miR-133a because they express very low levels of this miR^16,19^. Treatment of NIH 3T3 cells with myofiber-derived exosomes for 48 hours significantly and reproducibly reduced Smarcd1 and Runx2 protein levels (Fig. 5A and B). The findings confirm that cargos transferred to recipient cells by exosomes derived from myofibers exert biological effects.

## Discussion

We investigated whether isolated skeletal muscle fibers release exosome-like nanovesicles when cultured *in vitro*. This approach extends prior work examining exosomes released by myoblasts or myotubes derived from C2C12 cells^7–12^, differentiating human skeletal myoblasts^14^ or present in preparations of skeletal muscle^13^. Full-length muscle fibers remain viable for at least 1 week in culture which affords an opportunity to examine their properties outside of the confines of an individual muscle, thus reducing confounding effects of other cellular elements that compose skeletal muscle that is blood vessels, connective tissue, nerves and fat. Dispersed fibers have been used to study a wide variety of properties of skeletal muscle, including membrane permeability^20^, calcium handling^21^ and sarcomere function^22^.

The findings presented above demonstrate that cultured myofibers release nanovesicles that possess many of the characteristic features of exosomes described in the literature^5,23^: sizes of 60 to 130 nm, a bilamellar membrane and positive immunogold staining for the tetraspanins CD63, CD81 and CD9^5,24^. The myofiber-derived nanovesicles were taken up by C2C12 myoblasts and contained microRNAs. The exosomes released by cultured myofibers could arise from the myofiber itself and/or from the satellite cells located between the sarcolemma and basement membrane surrounding the fiber. Given the much greater mass of the muscle fiber and the more limited number of satellite cells it is likely that the majority of exosomes evaluated in our studies were derived from the skeletal muscle fiber.

Taken together, our data indicate that intact muscle fibers release exosomes and provide a means to study the properties of exosomes released by such fibers from healthy and diseased muscle without contamination from exosomes released from other cell types present in the muscle.

The exosomes contained microRNAs that were also present in the parent myofibers, with miR-133a being by far the most abundant. Consistent with prior profiling studies in different cell types^25,26^, there appeared to be some selectivity in packaging of miRs in fiber-derived exosomes. For example, the relative levels of miR-720 were enriched in exosomes as compared to those present in the fibers, while miR-1 was, to a degree, excluded.

The exosomes released by cultured muscle fibers not only could be taken up by target C2C12 cells but also could downregulate the expression of Smarcd1 and Runx2 proteins in NIH3T3, providing evidence that such exosomes can signal through their cargo to nearby cells in a paracrine manner. In the case of Smarcd1 and Runx2 proteins, their downregulation by fiber-derived exosomes was likely to be mediated by miR-133a because both have been reported as validated targets of this miR^16,17^, it is the most abundant miR found in these exosomes, and evidence exists that only the most highly-expressed miRs regulate target mRNAs^27^.

Smarcd1 is involved in skeletal muscle myogenesis^15^, possibly by binding MyoD^28^, which raises the possibility that myofiber-derived exosomes may modulate Smarcd1 protein levels of nearby satellite cells and/or other myogenic precursors and influence their biology. In a healthy muscle, myogenic precursors are largely comprised of quiescent satellite cells which reside between the sarcolemma and basement membrane, placing satellite cells very close to muscle fibers. Thus, satellite cells are bathed in extracellular fluid enriched in substances secreted by the fiber including exosomes. These observations raise the possibility that the increased miR-206 present in exosomes from denervated fibers may play a role in the activation and/or proliferation of satellite cells known to occur after nerve transection^29–31^. In addition, muscle levels of myomirs have been shown to be altered by older age, gender and testosterone levels^32^. Advanced age reduces satellite cell proliferation^33^ leading to the question of whether age-related changes in exosomal myomir cargo contributes to this satellite cell abnormality. Future studies are needed to address these provocative questions.

The possibility that miRs in exosomes released by either healthy or paralyzed muscle might have distant effects should also be considered. In support of this proposal, and as noted above, changes in miR profiles of exosomes derived from skeletal muscle have been suggested to regulate pancreatic β-cell gene expression^13^. Because myofiber-derived exosomes can modulate levels of Runx2, an osteoblast differentiation factor, the possibility that such exosomes could also influence the fate of mesenchymal stromal cells, and thus bone biology, must be considered.

The most dramatic denervation-related change in miR expression levels was the marked increase in both myofibers and exosomes of miR-206. miR-206 is a skeletal muscle specific miR that is greatly upregulated during myoblast differentiation and promotes satellite cell differentiation by regulating the expression of HDAC4^34^, as well as that of Pax3, Pax 7, utrophin, DNA polymerase-alpha and connexin 43^35–37^. Several lines of evidence indicate that miR-206 is involved in muscle regeneration in both mouse and rat models^38–40^ although there is also evidence implicating miR-206 in growth-inhibitory and/or muscle atrophy programs^18^. Regardless of whether miR-206 promotes or reduces atrophy, the marked increase in miR-206 in exosomes released by denervated muscle fibers has obvious implications for inter-cellular signaling during denervation atrophy. Since potential targets of miR-206 include molecules such as BDNF, NGFR, IGF-1 and IGFBP-5, it has been suggested that miR-206 could also be involved in the regulation of muscle mass and in synapse formation during re-innervation^38,41^. Thus, upregulation of miR-206 in fiber-derived exosomes after denervation could be a teleologic strategy to aid in local muscle repair by providing readymade miR-206 in a paracrine manner to neighboring cells. The large increase in miR-206 observed in exosomes produced by denervated myofibers could have significant effects on other types of recipient cells such as motor nerve terminals at the motor end plate and neighboring capillaries, arteries or veins and, extending the biological action of this myomiR, on non-muscle cells. For example, miR-206 could also affect bone because it inhibits osteoblast differentiation via downregulation of connexin 43^42^.

Mechanisms by which denervation drives alterations of miR expression profiles are poorly understood. MyoD, a critical myogenic differentiation factor from the basic helix-loop-helix family of transcription factors, drives expression of several myomiRs^43^ and is persistently elevated in denervated muscle^44^. Thus, increased MyoD-induced transcription may explain some changes in miR expression in denervated muscle, such as upregulation of miR-206, but seems less likely to contribute to reduced expression of miR-1 or miR-133. Critical upstream signals responsible for denervation atrophy involve activation of non-classical NF-kB signaling through p65 and cRel^45^ which appears to be downstream of the *de novo* sarcolemmal expression of connexin 43 and 45 hemichannels^20^. Further study will be required to understand how these signaling pathways directly or indirectly modulate transcription and stability of miRNA transcripts.

The clinical significance of our findings remains speculative. There are several reports in which blood miR levels have been proposed as a biomarker for a variety of diseases including those affecting skeletal muscle^46,47^. The myomirs have been identified in the circulation where their levels have been shown to change with exercise^48–51^ or muscular dystrophies^46,47^. The origin of circulating myomirs is not well understood. In one study, extracellular vesicles isolated from blood by density gradient separation were positive for the markers CD81, TSG101 and alpha-sarcoglycan and contained miR-206, suggesting that they arose from cells of the myogenic lineage^52^. In a separate study, a bout of exercise was found to increase the number of small extracellular vesicles in blood^53^. The possibility that myofibers signal to distant targets via release of exosomes is an interesting topic for future studies.

In summary, our findings provide direct support for the conclusion that myofibers release exosomes that contain microRNA as cargo and that exosomal miR profiles are altered by denervation. The data above supports the conclusion that myofiber-derived exosomes modulate protein levels of key factors in myogenic or osteogenic differentiation of mesenchymal progenitor cells, and likely other cell types, pointing to the exciting possibility of a role of such exosomes in paracrine regulation by myofibers of nearby cells. Our findings also raise the possibility that myofiber-derived exosomes are an important source of circulating myomirs and extracellular vesicles, providing support for the use of circulating myomiRs levels as biomarkers for muscle health and disease.

## Materials and Methods

### Animals

Male C57B/6 J mice weighing 25 g were obtained from Jackson Labs (Bar Harbor, ME) and housed in rooms with 12:12 hour light: dark cycles and controlled temperature and humidity. Animals were provided food and water *ad libitum*. Denervation was performed as previously described with some modifications^20^. The animals were anesthetized by inhalation of isofluorane, hair was removed with a clipper and the skin was cleaned with betadine solution and ethanol. A small incision was made just posterior to the head of the left femur and the sciatic nerve was exposed by careful dissection. A 2 mm piece of the nerve was removed and the wounds were closed with suture and surgical glue. Animals were administered carprofen for 3 days postoperatively. Some animals received a sham nerve transection in which the nerve was exposed but not manipulated. At 7 days after nerve or sham transection, animals were anesthetized by inhalation of isofluorane and hindlimb muscles were removed by careful dissection, minimizing any pulling on the muscle. All studies with animals were approved by the IACUC at the James J. Peters VA Medical Center and were carried out in accordance with the NIH Guide for Care and Use of Laboratory Animals.

### Isolation and culture of skeletal muscle fibers

Muscle fibers were isolated and maintained, as previously described^54^. Briefly, muscle tissue (gastrocnemius, plantaris, EDL and soleus, or EDL alone, as indicated in the figure legends) was removed from mouse hindlimbs as above described and immediately digested with 400 U/ml of collagenase Type I (Worthington) in DMEM-F12 medium (1:1) (ThermoFisher) at 37 °C for 45–60 minutes, depending on the collagenase lot. The digested tissue was transferred to DMEM/F12 (1:1) supplemented with Penicillin/Streptomycin/Amphotericin B (Antibiotics-Antimycotic,ThermoFisher) and 15% exosome-depleted horse serum prepared by overnight centrifugation at 100,00 × g^55^. Digested muscle tissue was triturated with a wide bore pipette to release single fibers. The released fibers were collected with a fire-polished glass pipette and transferred to a pre-warmed dish containing the above medium. The process was repeated until the required number of fibers was collected. Fibers were passaged 2–3 times in fresh pre-warmed medium to remove debris and cultured for 48 hours. Typically, 150–600 fibers were cultured with a survival of about 90%.

### Isolation of nanovesicles

Nanovesicles were isolated by differential centrifugation^55^. The fiber conditioned medium was collected and centrifuged at 1800 rpm for 30 minutes, followed by centrifugation at 10,000 rpm for 45 minutes. The resulting supernatant was centrifuged at 100,000 × g for 70 minutes (Beckman Coulter Optima, type SW-41 rotor). The pellet containing nanovesicles was resuspended in PBS and ultracentrifuged, as described above.

### Extraction of RNA

Total RNA was isolated from the cultured fibers and corresponding nanovesicle preparations using the miRNeasy microRNA isolation kit (Qiagen, Germantown, MD). Briefly, fibers pellets or nanovesicles prepared as above were extracted with Qiazol reagent (Qiagen) and the RNA was further purified according to the protocol provided by the manufacturer (Qiagen). In the case of nanovesicles, cel-miR-39 (8 pg) (Qiagen) was added to the Qiazol extract for internal normalization prior to miR purification.

### microRNA profiling

The profile of microRNA levels present in skeletal muscle fibers was examined using nCounter mouse miRNA arrays (NanoStrings Technologies, Seattle, WA) which survey the expression levels of 578 mouse microRNAs. miRNA profiling was performed through the POP service of the manufacturer (NanoStrings Technologies). Raw data were normalized using the average intensity of the 100 most highly expressed miRNAs. Average counts of negative controls +2 standard deviations were subtracted from the individual counts. miRs for which counts were greater than or equal to 100 after these corrections were included in subsequent analysis and fold-change in expression levels between sham and denervated samples was calculated.

### Quantitative RT-PCR

Total RNA was reverse transcribed using a pool of the specific primers for the miRs of interest provided with the TaqMan miR assays. The primers were pooled and diluted to a final 1:100 dilution. Reverse transcription was performed with the TaqMan MicroRNA Reverse transcription kit (Life Technologies-ThermoFisher) according to the protocol provided by the manufacturer (Life Technology protocol #4465407). Due to the low amount of starting material (22 ng of RNA for fibers and 3 μl/20 μl of RNA for the nanovesicles) a pre-amplification step was performed with the pre-AMP Master mix according to the manufacturer’s protocol (Life Technologies protocol #4465407). The pre-AMP product was diluted 1:8 and 0.8 μl of preamplification product was amplified using specific miR TaqMan assays and the TaqMan Universal PCR Master mix (Life Technologies-ThermoFisher) in a 10 μl reaction. Expression levels were normalized against U6sn for muscle fibers, and cel-miR-39 for nanovesicles. Relative expression was calculated with the 2^−∆∆Ct^ method^56^ using the sham-denervated samples as a control.

### Size distribution of nanovesicles released by cultured muscle fibers

Nanovesicle tracking analysis was performed with a NanoSight LM10-HS instrument (Malvern Instruments, Westborough, MA). An aliquot of the nanovesicle preparation (derived from 700 fibers) was diluted 1:25 in PBS and loaded onto the NanoSight instrument after calibration with 100 μm polystyrene latex microbeads (Malvern Instruments). Five, 60 seconds videos were recorded at a camera level set at 14. The videos were analyzed with the NS500 software to determine the particles size distribution and concentration using the following parameters: auto-blur, track length and size and a threshold detection of 10.

### Electron Microscopy

#### Negative staining of exosomes with uranyl acetate

Nanovesicles were isolated from the conditioned cultured medium of mouse calf muscle fibers (about 240 fibers), as described above. The 100,000 × g pellet was suspended in 2% paraformaldehyde in PBS and 5-μl aliquots deposited for 20 minutes on formvar- and carbon- coated 200-mesh copper grids (Electron Microscopy Sciences, Hatfield, PA). Samples on grids were then fixed with 1% glutaraldehyde for 5 minutes, and negatively stained with uranyl acetate-oxalate solution for 5 minutes followed by embedding in methyl cellulose as described^55^. Samples on grids were viewed using a Hitachi H7000 electron microscope (Tokyo, Japan) at 75 kV. The electron microscope was equipped with an AMT Advantage HS digital camera (Danvers, MA, USA) and micrographs were digitally recorded.

#### Immunogold labeling of exosomes

Five-μl aliquots of exosomes suspended in 2% paraformaldehyde in PBS were deposited for 20 minutes on formvar- and carbon-coated 200-mesh copper grids and immunogold labeled as described^55^ with the following modifications. Blocking media consisted of PBS containing 0.1% gelatin and 0.1% bovine serum albumin (PBS+) for 10 minutes followed by 5% goat serum in PBS+ for 20 minutes. Samples on grids were incubated with primary antibodies diluted 1:100 in 1% goat serum in PBS+ for 1 hour then with secondary antibodies conjugated to 10-nm immunogold (Electron Microscopy Sciences, Hatfield, PA) diluted 1:25 in PBS+ for 1 hour. Immunogold-labeled samples were post-fixed with 1% glutaraldehyde for 5 minutes, and negatively stained with uranyl acetate-oxalate solution for 5 minutes followed by embedding in methyl cellulose. Primary antibodies against CD63, CD81 and CD9 were purchased from System Biosciences (SBI, Palo Alto, CA). Samples on grids were imaged with the electron microscope as described above.

### Uptake of nanovesicles by C2C12 myoblasts

To study uptake, nanovesicles isolated from cultures of ~300–400 fibers were labeled with the fluorescent dye PKH67 (Sigma Aldrich, St Louis, MO). The 100,000 × g pellet was resuspended in 150 μl diluent C (Sigma Aldrich, St Louis, MO) and the suspension diluted with an equal volume of diluent C. The PKH67 dye stock was diluted 60-fold in 250 μl diluent C (Sigma Aldrich, St Louis, MO) and the nanovesicle preparation or an equal volume of PBS was added to the dye. After incubation at room temperature for 5 minutes, an equal volume of 1% BSA was added to quench the reaction. The mixture was diluted with PBS and centrifuged for 70 minutes at 100,000 × g. The pellet was resuspended in PBS and centrifuged as described above.

C2C12 myoblasts were seeded in 4 well slide chambers and allowed to reach confluence. The labeled vesicles or control dye were resuspended in 300 μl of exosome-depleted DMEM/2% horse serum and added to C2C12 myoblasts. After incubation for 5 hours, the cells were fixed in 4% paraformaldehyde in PBS for 15 minutes and washed with PBS. The cells were permeabilized with 0.1% Triton in PBS for 3 minutes, washed with PBS and blocked with 10% normal goat serum (Sigma Aldrich) in 50 mM Tris HCl buffer pH 7.4, 0.15 M NaCl (TBS) for 30 minutes. Alexa-568 labeled phalloidin (Life Technologies-ThermoFisher) was added at 1:1000 dilution in blocking solution for 30 minutes. Nuclei were counterstained with 4,6-diamidino-2-phenylindole (DAPI). The slides were mounted in Fluorogel mounting medium (Electron Microscopy Sciences, Hatfield, PA) and imaged with a Zeiss confocal microscope. Images were processed with Adobe Photoshop CC.

### Treatment of NIH 3T3 cells with fiber-derived exosomes

Exosomes were prepared as above, resuspended in PBS and the protein concentration determined by the Micro BCA assay (Pierce-ThermoFisher). NIH3T3 were seeded in 24 wells plates (6–7 × 10^4^ cells/well) and cultured in DMEM supplemented with 10% heat-denatured calf serum and with Antibiotics-Antimycotic (ThermoFisher) and depleted of exosome by ultracentrifugation as described above. Exosomes were added at a concentration of 4 μg protein/ml medium for 48 hours, while control wells received the same volume of PBS. Cells were washed twice with cold PBS and lysed with 10 mM Na phosphate pH 7.4, 150 mM NaCl, 2 mM EDTA, 1% Triton X-100, 0.5% Na deoxycholate, 0.5% SDS supplemented with protease and phosphatase inhibitor cocktails (Sigma Aldrich, St Louis, MO). Lysates were briefly sonicated, centrifuged at 14,000 rpm for 15 minutes at 4 °C and the supernatant saved. Protein concentration were determined with the BCA reagent (Pierce-ThermoFisher). Proteins (20–25 μg) were separated by SDS PAGE, and the gels blotted onto a PVDF membrane. The membrane was blocked with 0.5% non-fat dry milk in Tris buffer saline (TBS) and incubated overnight at 4 °C with the primary antibody diluted in blocking solution. The blot was incubated with the appropriate HRP conjugated secondary antibody (1:5000–1:7500, GE Healthcare Life Sciences, Marlborough, MA) in blocking solution, exposed to ECL Prime Western Blot detection reagent (GE Healthcare Life Sciences) and imaged with Amersham Imager 600 (GE Healthcare Life Sciences). The following antibodies were used: anti- Smarcd1 rabbit polyclonal antibody (1:500 dilution; AB83208, Abcam, Cambridge, MA), anti-Runx2 mouse monoclonal antibody (1:500 dilution; Santa Cruz Biotechnology, Dallas, TX,) and a mouse monoclonal antibody against GAPDH (1:4000 dilution; GeneTex, Irvine, CA) as loading control. Quantification of the bands was performed with Image Quant software (GE Healthcare).

### Data Analysis

All values are expressed as means +/− SD. Statistical analysis was performed with Graph Pad Prism v 7.0 software. An unpaired, two-tailed t-test was used to test the significance of differences between means. P < 0.05 was considered significant.

## Electronic supplementary material

Figure S1

Table S1

Table S2
