# Supplementary material for: Denervation-related alterations and biological activity of miRNAs contained in exosomes released by skeletal muscle fibers
Source: Sci Rep. 2017 Oct 16;7:12888. doi: 10.1038/s41598-017-13105-9 (PMC5643439; doi:10.1038/s41598-017-13105-9)
Supplement: Supplementary file 1 — Figure S1 [file 41598_2017_13105_MOESM1_ESM.doc]

Supplementary Figure:

**Denervation-related alterations and biological activity of miRNAs contained in exosomes released by skeletal muscle fibers**

Rita De Gasperi^1,7,8^, Sayyed Hamidi^2^, Lauren M. Harlow^1^, Hanna Ksiezak-Reding ^6^, William A. Bauman^1,2,3,4^, and Christopher P. Cardozo^1,2,3,4,5*^

^1^ National Center for the Medical Consequences of Spinal Cord Injury and ^2^ Medical Service, James J. Peters VA Medical Center, Bronx, NY

^3^ Departments of Medicine, ^4^ Rehabilitation Medicine, ^5^ Pharmacologic Science, ^6^ Neurology,

^7^ Psychiatry, and ^8^ Friedman Brain Institute, Icahn School of Medicine at Mount Sinai, New York, NY

*Corresponding Author:

Christopher Cardozo

National Center for the Medical Consequences of SCI

James J. Peters VA Medical Center

130 West Kingsbridge Road

Bronx, NY 10468

Tel 718-584-9000 x 1828

Fax 718-741-4675

chris.cardozo@mssm.edu


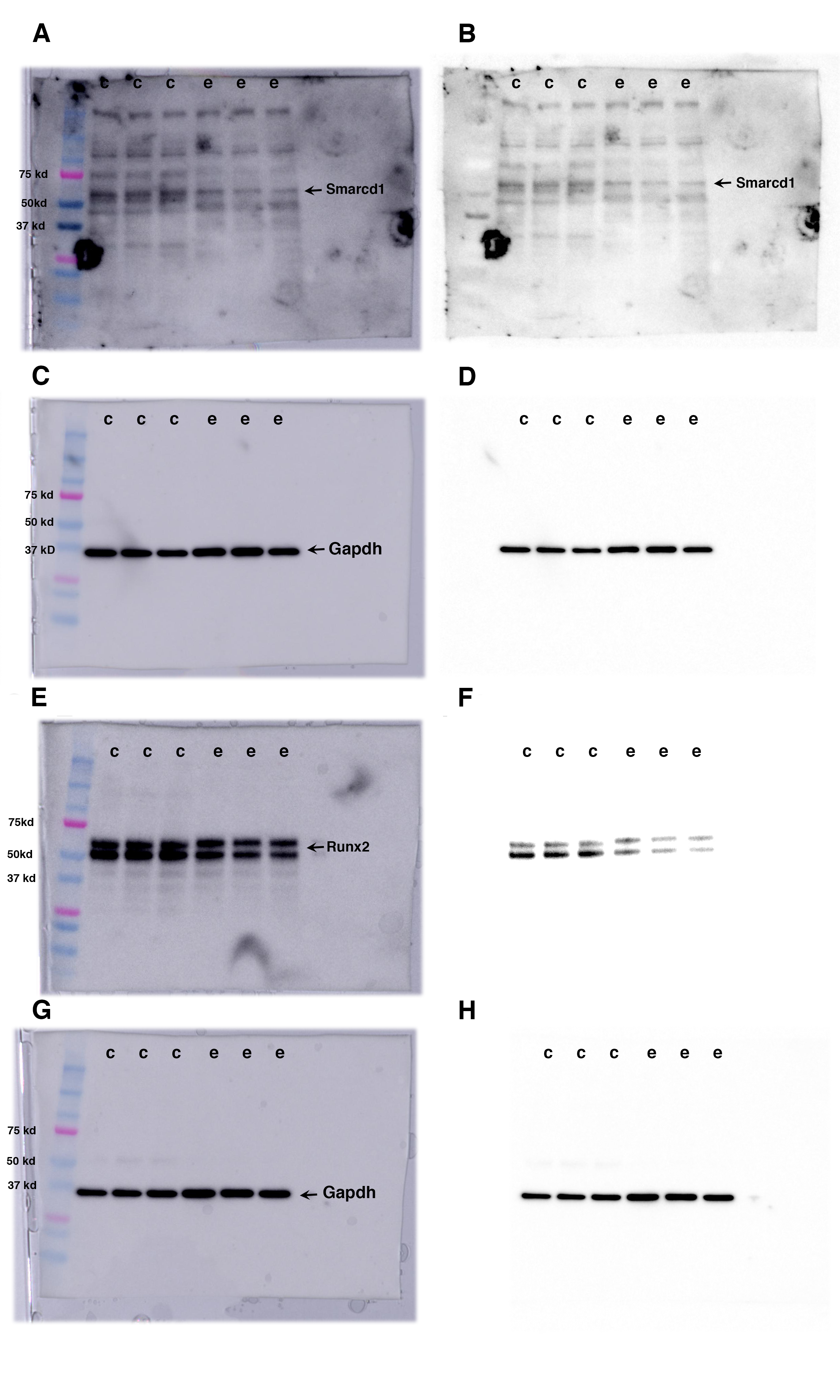


**Figure S1**: **Full-length blots of data shown in Figure 5A.** The panels are raw images of exposures obtained with an Amersham 600 Imager. The merged chemiluminescence+ colorimetric images generated by the Imager 600 (panels **A**, **C**, **E** and **G**) are shown to visualize the molecular weight markers along with the chemiluminescence signal. For each target the chemiluminescence only ( **B, D, F and H**) and merged images represent different exposures of the same blot. Smarcd1 (**A, B**); Gapdh probing of Smarcd1 blot as loading control (**C, D**); Runx2 (**E, F**); Gapdh probing of Runx2 blot as loading control (**G, H**). **c**, control; **e**, exosomes
